# Supplementary material for: Are carbon emissions trading and green financial instruments synergistic? -Comprehensive quantitative research based on content analysis
Source: PLoS One. 2024 Mar 7;19(3):e0298601. doi: 10.1371/journal.pone.0298601 (PMC10919601; doi:10.1371/journal.pone.0298601)
Supplement: S1 Data — (ZIP) [file pone.0298601.s001.zip › Data/地级市Z_F工作报告.zip!/╦─┤¿/─╧│Σ╩╨/└┤╫╘╣1⁄2╓┌║┼í╛┬φ┐╦╩2╛▌═°í┐_2015.docx]

各位代表：

我代表市人民政府向大会报告工作，请予审查，并请市政协委员和其他列席会议的同志提出意见。

一、2014年工作回顾

过去的一年，是南充发展史上非常特殊、非常困难的一年。面对复杂严峻的宏观经济形势和南充炼油厂全面关停等因素影响，我们认真贯彻省委、省政府和市委的决策部署，着力改革开放、投资拉动、产业支撑、城乡统筹和民生改善，全市经济社会持续健康发展。全年实现地区生产总值1432亿元、增长7.2%，一般公共预算收入76.6亿元、增长16.8%。

　（一）重点改革有序实施。农村产权制度改革稳步推进，全市农村集体土地所有权、集体建设用地使用权确权登记颁证工作基本完成，顺庆、南部、仪陇农村产权流转交易中心挂牌运行。地方金融改革步伐加快，仪陇信用联社改制成为川东北第一家农商行，营山农商行挂牌营业；生猪价格指数保险、农村土地流转收益保证贷款和农民财产性增收等试点工作有序开展。投融资体制改革扎实推进，成功组建南充工业发展投资有限公司；积极拓宽融资渠道，实现直接融资82亿元、仓储融资12亿元。公立医院改革进展顺利，成功申报为第二批公立医院改革国家联系试点城市，市域公立医院药品加成全面取消，分级诊疗制度全面推行。政府自身改革不断深化，新一轮政府机构改革基本完成；累计取消、调整、下放行政审批事项130项，市级层面核准事项减少50%以上；大力推行工商注册制度便利化，全市新登记企业增长30%。

（二）投资保持合理增长。全市完成全社会固定资产投资1244.5亿元、增长12.8%，其中150个市列重点项目完成投资553亿元。政府性投资稳步增长，全市完成270.9亿元、增长10.5%。兰渝铁路南充段路基基本成型，南充经开区铁路专用线、南充港都京作业区多用途码头和巴南广高速公路等项目进展顺利，火车北站配套工程、嘉陵江航运配套工程、高坪机场航站楼改扩建工程启动建设；解元水库全面竣工，九龙潭水库大坝主体完工，升钟水库灌区二期、油房沟水库加快建设。民间投资快速增长，全市完成703.2亿元、增长27%。欧尚、奥特莱斯等国内外知名企业相继入驻，吉利集团10万台新能源商用车和5万台燃气发动机生产项目落户南充，全市招商引资到位资金468.3亿元。金融支持力度明显加大，全市各项贷款余额首次突破千亿大关、达到1059亿元，比年初增加216亿元、增长25.6%。国际交流合作取得新成效，成功举办“2014四川（南充）·法国周”活动，与法国香槟—阿登大区政府签订6个方面的合作协议，中法农业科技园、中法文化创意产业园正式挂牌并启动建设。

（三）三次产业稳步发展。着力促进工业减损止滑，规模以上工业增加值增长6%。晟达新材料系列项目公辅工程全部完工，首套装置精对苯二甲酸开始单机试车；康美药业、富安娜二期等项目竣工投产，鑫达新材料、创越炭材料等项目进展顺利。着力促进农业增产增效，南充国家现代农业示范区4个“百公里现代循环农业示范带”实现全覆盖，在全国农业改革与建设试点示范区绩效考评中位列全国第5、中西部第1；以西充县为核心区、三区为示范区的农业科技园区成功申报为国家级农业科技园区；全市实现粮食总产量304.9万吨，连续12年居全省第1。着力促进服务业提档升级，实现社会消费品零售总额593.4亿元、增长13.4%。南充现代物流园传化公路港等项目加快建设，川北农产品批发市场、阆中（义乌）商贸城、南部（亿联）五金建材城等项目投入运营；积极推进旅游业与工农业互动融合，乡村旅游蓬勃发展，工业旅游日渐兴起，全市实现旅游总收入253.8亿元、增长20.8%。

（四）城乡建设统筹推进。全市城镇化率提高1.5个百分点、达到42.4%。城镇布局不断优化，《南充市城市地下空间开发利用规划》和仪陇县城市总体规划修编通过省评，阆中市城市总体规划批准实施，西充县纳入全省全域规划试点县；新建“幸福美丽新村”65个、新村聚居点196个。城镇功能不断完善，中心城区北部新城、南部产业新城和下中坝片区、清泉坝片区加快建设，市规划展览馆、科技馆、地方志馆和青少年活动中心启动建设；阆中、仪陇、蓬安跨江大桥建成通车，南部奥体馆建成开馆，西充文博馆主体完工；阆中成功入选全国首批国家新型城镇化综合试点县（市）。生态建设不断加强，全市森林覆盖率达到39.6%，新增城镇绿地面积360公顷、公园绿地面积110公顷；西充河Ⅲ类水质达到329天，生活污水集中处理率和生活垃圾无害化处理率分别达到84%、89.6%，单位生产总值能耗下降4.2%。

　（五）人民生活持续改善。大力实施“十项民生工程”和20件民生实事，民生支出占一般公共预算支出比重达70.6%。全市城镇居民人均可支配收入、农民人均纯收入分别增长10.5%、11.8%。新增城镇就业8万人，失业人员再就业1.6万人。在全省率先整合新农保和城市居民养老保险，率先建立丧葬补助费和一次性抚恤金制度；成功推行城镇医保付费制度改革和居民门诊统筹，实现市内职工医保实际缴费年限互认。开工建设保障性住房和棚户区改造安置住房10.7万套，建成面向农民工出租的公租房1260套。大力实施扶贫攻坚，13.2万农村贫困人口脱贫解困。完成农村薄弱学校校舍改造43.5万平方米，新（改扩）建幼儿园41所，张澜职业技术学校成功创建为技师学院。市中心医院门诊住院大楼、川北医学院附属医院新院区门诊楼和市中医院综合楼投入使用，20个乡镇卫生院、100个村卫生室全面建成。积极创建国家公共文化服务体系示范区，基本实现市、县、乡、村四级公共文化阵地全覆盖。新建通乡通村公路2500公里、公路安保设施3531公里。深入推进社会治安综合治理，大力开展食品药品和安全生产专项整治，人民群众安全感进一步增强。

与此同时，国防动员、人民防空和双拥优抚工作扎实推进，行政监察、审计监督、国资监管、统计调查、检验检疫、计划生育、质监、海关、气象、档案、保密、国安、邮政、外事侨务对台等工作取得新成绩，民族宗教、地方志、老龄、妇女儿童、残疾人、红十字会等工作发挥了积极作用。

各位代表，过去一年，在多重挑战集中显现、多重困难相互交织的严峻形势下，全市上下的团结奋斗弥足珍贵，持续健康的发展态势来之不易。在此，我代表市人民政府，向全市各族人民，向各民主党派、工商联、无党派人士和人民团体，向驻市解放军、武警部队官兵、政法干警和各界朋友表示衷心的感谢，并致以崇高的敬意！

各位代表，全面回顾去年工作，我们也清醒地看到，发展中还存在许多困难和问题：部分经济指标增速回落，稳增长难度持续加大；产业结构性矛盾突出，发展方式低效粗放，创新能力严重不足；投资高位增长难以为继，特别是工业投资总量小、占比低，新旧增长动力接续出现断档；政府性债务包袱沉重，偿债压力日益凸显；城乡困难群众量大面宽，扶贫攻坚任务艰巨；政府系统“四风”问题依然不同程度存在，部分工作人员慢作为、怕作为、不作为，有的甚至无视党纪国法，以权谋私，行贿受贿，堕入违法犯罪深渊。这些问题不容回避，我们将本着对党和人民高度负责的态度，认真对待，强力解决。

二、2015年工作安排

2015年是全面完成“十二五”规划的收官之年，也是南充攻坚克难、转型发展的关键之年。今年政府工作的总体要求是：紧紧围绕“四个全面”战略布局，认真贯彻党的十八届三中、四中全会、省委十届四次、五次全会和市委经济工作会议精神，主动适应经济新常态，突出发展第一要务，深入实施“三大发展战略”，统筹推进稳增长、促改革、调结构、惠民生、防风险，努力实现经济持续健康发展和社会和谐稳定。

全市经济社会发展的主要预期目标是：地区生产总值增长7.5%，全社会固定资产投资增长10%，一般公共预算收入增长7.5%，规模以上工业增加值增长8%，社会消费品零售总额增长12%，城镇居民人均可支配收入增长10%，农村居民人均可支配收入增长10.5%。

围绕上述目标，重点抓好六个方面工作：

（一）全力支持实体经济，统筹施策稳定经济增长

　　促进投资稳步增长。牢牢抓住项目“牛鼻子”，准确把握国家产业政策和投资方向，科学编制“十三五”规划，确保一批打基础、利长远的重大项目挤进国、省项目库。采取特许经营、政府与社会资本合作等多种方式，撬动民间资本参与基础设施、公用事业等领域的重点项目建设。注重发挥财政投资的引领作用，扩大技术改造、科技创新等领域的投资，不断提升产业投资比重。坚持挂图作战，倒排工期，强力推进150个市列重点项目建设，带动完成全社会固定资产投资1370亿元以上。

精心呵护实体企业。引导金融机构加大支持实体经济力度，鼓励金融服务创新，力争贷款余额增长20%以上；设立企业应急转贷资金，完善企业融资风险分担机制，多方联动帮助企业缓解融资难、融资贵问题。全面落实结构性减税、普遍性降费等财税支持政策，抓紧建立涉企收费清单，严格规范行业协会、中介组织收费，切实减轻企业负担。积极争取直购电、丰水期富余电量消纳等用电优惠政策，加大对用气大户直供的协调力度，降低企业生产成本。推行领导干部对口联系重点项目、困难企业制度，采取“一企一策”，切实帮助企业解决具体问题。

有效扩大消费需求。 坚持以新产业、新产品、新网络促进消费，增强消费对稳增长的基础性作用。大力发展电子信息、网络通讯、健康养老、节能环保等产业，培育新的消费热点。围绕优势产业、重点企业、特色产品，深入实施质量强市和商标品牌战略，大力发展节会经济，扩大“南充造”产品市场占有率。加快城乡流通体系建设，优化城市商业网点布局，完善农村电商配送网络，建成南充鲜活农产品网上交易平台。

（二）深入推进改革开放，持续增强经济发展动能

　　不断深化重点改革。 加快推进农村产权制度改革，有序开展确权登记颁证工作，全面建成九县（市、区）农村产权流转交易中心，积极开展农村集体资产股份制改造试点，探索开展财政支农资金形成资产股权量化改革试点。大力推进地方金融改革，以优化股权结构、人才结构为核心，加快农村信用社改制步伐，确保阆中、西充农商行开业，三区农信社组建农商行通过中国银监会审批；推进政府性融资平台公司转型升级；加快发展村镇银行、农村资金互助社等新型金融组织，完善农村普惠制金融体系。稳妥推进国资国企改革，鼓励各类投资者参与国有企业改制重组，大力发展混合所有制经济；成立市政府国有企业监事会，建立规范的监事会派驻企业制度。积极推进财税体制改革，进一步完善市区事权、财权相匹配的体制；充分挖掘收入潜力，着力优化支出结构，提升财政资金使用效益。扎实推进公立医院改革，大力引进社会资本参与公立医院发展，建立健全基层首诊、双向转诊、急慢分治的就医秩序，不断完善公立医院管理体制和运行机制。

务实开展招商引资。 始终保持招商引资高位态势，围绕重点产业，瞄准目标企业，大力开展产业招商、沿链招商，力争全年到位资金460亿元以上。积极营造诚实守信的政务环境，认真落实差别化招商引资政策，加大对新引进重点产业项目和创新型项目的支持力度。密切与珠三角、长三角、环渤海湾等发达地区的联系，主动承接产业转移。

全面深化区域合作。主动融入“一带一路”和“长江经济带”等开放大格局，积极申建保税物流中心和国家开放口岸、进口指定口岸。大力实施川东北区域发展规划，优先推进区域基础设施互联互通，积极促进科教文卫资源共享、优势产业一体化布局。巩固提升对口援建天全县灾后重建成果，促进对口援建向对口合作延伸。继续深化与法国的合作，进一步扩大与欧盟国家及台湾地区的交流合作。

（三）大力实施创新驱动，充分激发创新创业活力

　　打造创新创业平台。坚持把南充创新驱动发展试验区作为推动创新创业的“试验田”，加快建设大学科技园、中法文化创意产业园和创新创业孵化产业园，扎实推进中法农业科技园、农业科技孵化中心建设。积极搭建协同创新平台，引导企业与高校、科研院所建立产学研联盟、技术转移中心、大学生实训基地；大力实施“互联网+”行动计划，筹划建设南充云计算中心，促进科技基础条件平台开放共享。鼓励搭建大众创业平台，支持三区和有条件的县（市）发展创客空间、新型孵化器等创业载体，利用现有产业园区标准厂房建设大学生创业园、返乡农民工创业园。

构建创新创业机制。发挥财政资金杠杆作用，设立创新创业投资引导基金，引导社会资金和金融资本支持创新创业。积极推动银科对接，利用中小企业私募债、资产证券化、银行间市场等拓展科技型企业融资渠道，鼓励金融机构为创新创业企业和人才提供特色金融产品和服务，力争成为全省科技小额贷款试点市。加强人才培养和引进，深化与西华师大、川北医学院等高校的战略合作，支持西南石油大学（南充校区）办成西部一流的应用型本科大学，积极引进领军人才、专业技术人才和创新创业团队，壮大创新创业队伍。

营造创新创业环境。深入推进商事制度改革，探索实行电子营业执照和全程电子化登记管理，推行“一址多照”、“先照后证”、“三证合一”，简化工商注册手续，进一步降低创新创业门槛。加强知识产权保护，整顿规范市场秩序，构建公平公正的市场环境。大力实施大学生创业引领计划，强化职业教育和技能培训，鼓励科技人员离岗创业并允许兼职取酬，激活创新创业主体，推动形成大众创业、万众创新的良好局面。

（四）积极推动转型发展，加快构建现代产业体系

　　着力打造工业发展平台。加快完善南充经开区基础设施和配套服务设施，确保铁路专用线、化工专用码头建成投入使用；加快推进晟达新材料系列项目建设，全力支持晟达公司延长产业链条，确保精对苯二甲酸等项目竣工投产；力争中瀛石化130万吨重油催化裂解、卓达新型建材等项目早日落地建设。加快建设南充清洁能源汽车产业园，开工建设吉利集团10万台新能源商用车和5万台燃气发动机生产项目，促进南充汽车公司转型升级发展。鼓励县（市、区）工业园区突出比较优势，聚焦主导产业，推动特色发展、错位发展、集群发展。

稳步提高农业综合效益。加快建设南充国家现代农业示范区20万亩高产高效生态产业基地；以引进技术和资金为重点，加快建设中法农业科技合作项目，扎实推进南充国家农业科技园区建设。积极培育各类新型农业经营主体，鼓励农地有序流转，促进适度规模经营。扎实推进升钟水库灌区二期建设，确保油房沟水库年内竣工，力争金鸡沟水库开工建设，牵头启动亭子口灌区前期工作；新增高标准农田20万亩、有效灌面4.1万亩。

提速发展现代服务业。 加快推进南充现代物流园重点产业项目建设，大力招引知名物流企业，积极创建国家级示范物流园。开工建设南充国际商贸城，着力打造区域商贸中心；规划建设市级综合性电商园区，支持西充县开展全国电子商务进农村综合示范试点。加快建设川东北金融中心，全力争取四川农村信用社异地灾备中心和金融后台服务基地项目落户南充。深入推进嘉陵江（南充段）生态文化旅游区建设，支持阆中古城创建国家级旅游度假区、仪陇朱德故里景区创建5A级景区。积极开展中国西部国际丝绸服装博览会前期工作，规划建设川东北会展中心。

（五）着力增强综合功能，不断提升城市宜居品质

　　强化交通枢纽建设。抢抓国家支持西部地区综合交通建设的重大机遇，力争汉巴南铁路纳入国家路网规划，确保兰渝铁路南充段和南充火车北站及阆中站、南部站建成投入使用；加快建设遂西、巴南广高速公路，开工建设南绵、营达高速公路，力争开工建设南充过境高速公路广南至南广段，加快推进南部定水经升钟至阆中思依、顺庆经蓬安至营山一级公路建设。扎实推进嘉陵江航运配套工程和南充港都京作业区配套工程建设。加快实施高坪机场航站楼改扩建工程。

统筹推进城乡建设。 坚持以中心城区为龙头，协调推进县城建设和村镇建设，加快完善“1＋3＋6＋N”的市域城镇体系，全市城镇化率提升1个百分点以上。中心城区以在建项目为重点，加快完善下中坝片区、清泉坝片区公共服务设施，有序推进北部新城、南部产业新城配套设施建设；大力实施“天网工程”，充分发挥数字城管系统平台作用，有效提升城市智能化管理水平。着力完善县城功能，加快推进19个国家级重点小城镇、16个省级试点（重点）小城镇、9个市级扩权镇建设，完成400个“幸福美丽新村”、630个新村聚居点建设，促进城镇化与新农村建设协调发展。

扎实抓好生态建设。加快实施重点环保项目，启动建设嘉陵江南充段绿色生态走廊和西充河流域生态景观长廊，新增城市公园3个、生态绿地210公顷，新建污水处理厂5个、垃圾处理场2个；抓好天然林保护、退耕还林、水土保持等生态工程建设。加强嘉陵江流域及重点小流域水污染防治，深入开展大气污染综合治理，全面推进农村面源污染治理和生活垃圾集中处理，力争成为全国餐厨废弃物资源化利用和无害化处理试点城市，积极推动旅游景区“厕所革命”。坚决淘汰落后产能，大力发展循环经济和节能环保产业，全面完成“十二五”主要污染物减排任务。

（六）切实解决民生短板，努力增进人民群众福祉

　　办好民生大事实事。扎实推进“十项民生工程”，确保完成投资114亿元以上。着力办好20件民生实事：改善农村通村公路600公里；解决55.85万农村人口及11.74万学校师生饮水安全问题；新开工改造城市危旧房棚户区26739户；改造2.1万户农村危旧房；向农民工定向出租1031套公租房；采用政府购买服务方式，为28.22万名困难家庭失能老人和80周岁以上高龄老人提供居家养老服务；将60岁以上城乡居民基础养老金提高到每人每月75元；落实4200名在校大学生和高校毕业生创业补助政策，新增城镇就业6万人、失业人员再就业1.1万人；提高城乡居民基本医疗报销水平；强化“老幼病孕”免费基本公共卫生服务；帮助500名贫困白内障患者实施免费复明手术；建设边远贫困地区农村教师周转房442套；对全市63所公办普通高中按生均200元补助办学经费；免除1.16万名民办中职学校三年级学生学费；避险搬迁安置1610户农户；对全市5280个村提供公共服务经费补助；建成县级残疾人服务中心5个；建设（改造）农村电网线路872千米，改造10千伏配电变压器241台；新（改扩）建乡镇卫生院30所、村卫生室400个；中心城区新建公交场站3个，新增（更新）公交车71辆。

强力推进扶贫解困。全面实施扶贫开发五年规划，着力抓好“五大工程、一大行动”，确保11.3万人实现脱贫解困。突出抓好产业培育和能力培训，变“输血式”救济为“造血式”帮扶。全面完成县级减贫规划和专项扶贫规划编制；加强贫困人口精准识别工作，建立互联互通的贫困人口和贫困村管理信息系统，不断完善动态管理机制；瞄准贫困村和贫困人口，实行一村一策、一户一策，努力提高扶贫工作精准度和实效性。

强化基本公共服务。继续加大教育投入，开工建设38所公办幼儿园，改造义务教育阶段薄弱学校242所，完成中小学校舍改造9万平方米。加快建设市中心医院下中坝新院区门诊医技楼和川北医学院附属医院新院区住院大楼；加大乡镇卫生院、村卫生室标准化建设力度，积极探索养老医疗一体化服务新模式。开工建设市博物馆，加快建设市规划展览馆、科技馆、地方志馆和青少年活动中心，全面完成国家公共文化服务体系示范区创建任务。完善城乡居民大病保险、城镇居民基本医疗和补充医疗保险、企业职工基本养老保险制度，开展农村区域性养老服务中心建设试点。

提高社会治理水平。 强化系统治理、依法治理、综合治理和源头治理，不断改进社会治理方式。加强全民普法教育，深入开展“法律七进”活动。健全社会稳定风险评估机制，落实涉法涉诉信访依法终结制度。严格落实“党政同责、一岗双责、齐抓共管、失职追责”安全生产责任，坚决遏制重特大事故发生。深化食品溯源和市场准入管理，切实保障人民群众“舌尖上的安全”。扎实推进“平安南充”建设，严厉打击各类违法犯罪活动，坚决整治“黄赌毒”、“黑拐抢”等突出治安问题，确保人民群众生命财产安全，全力维护社会和谐稳定。

三、政府自身建设

从严治党务必从严治政，从严治政务必治之于行。我们将以高度的政治自觉、坚定的政治立场、鲜明的政治态度，知耻而后勇，深刻汲取南充近年腐败案件的沉痛教训，严格按照习近平总书记“三严三实”、省委“八个务必从严”和市委“三个带头、四个管好”的要求，切实加强“五型政府”建设，着力净化政治生态，重塑南充形象。

　（一）推行民主施政，全力打造开明开放政府。全面推行行政决策公开，扩大市政府全体会、常务会、专家论证会、决策评估会的社会参与度，完善重大行政决策公众咨询制度。深入推进市政府部门、公共企事业单位、乡镇（街道）、村（社区）政务公开标准化建设，切实加大行政审批、财政资金使用、公共资源配置、价格和收费信息等重点领域的公开力度。推行政府例行新闻发布制度，开发建设市政府门户网站手机客户端，构建政务微博、微信与政府网站一体化模式。

（二）强化责任担当，努力提高政府执行效能。坚持“一事一主体、一主体一责任人”，实行部门责任公开承诺制，落实重点项目、重点工作跟踪督办和责任追究制，着力构建逐级抓落实的责任体系。切实加强政府绩效管理，实行绩效评估与财务预算、项目经费管理相结合，继续推行社会公众满意度测评，不断增强绩效评估的激励和约束功能。深入开展“庸懒散浮拖”专项整治，坚决纠正慢作为、怕作为、不作为，推动形成言必责实、行必责实、功必责实的良好风气。

　（三）增强服务意识，大力营造优良政务环境。建立健全行政权力清单，对现有行政职权进行清理调整、依法审核确认，全面取消非行政许可审批事项，清理减少行政事业性收费。探索推行政府责任清单，进一步厘清市、区政府及部门的职责权限，做到权责匹配，防止缺位失位。抓紧制定企业投资负面清单，实行核准目录外企业投资项目不再审批。加快推进政务服务网建设，大力推行县、乡政务服务中心标准化建设，全面建成省、市、县三级联动行政审批网和市、县、乡三级联动便民服务网。

（四）践行法治原则，着力规范行政权力运行。坚持法之必行、有法必依，切实做到法无授权不可为、法定职责必须为。自觉接受人大及其常委会的法律监督、工作监督和政协的民主监督。坚持把合法性作为行政决策的第一要件，严格执行重大行政决策程序规定，降低决策风险，避免决策失误。完善行政执法与刑事司法衔接机制，推进信息共享平台建设。积极探索跨部门、跨行业综合执法，建立健全执法全过程记录制度、行政执法裁量权基准制度，切实做到规范公正文明执法。

（五）坚持标本兼治，强力推进党风廉政建设。坚决贯彻从严治党、从严治政各项规定，严格落实党风廉政建设责任制，针对政府系统近年来发生的违纪违法案件，深刻反思，举一反三，切实加强政府系统思想政治建设和反腐倡廉建设。重点围绕财政资金使用、工程建设、土地出让、征地拆迁和招投标等领域，建章立制，逗硬落实，有效防止权力失控。严格执行中央“八项规定”和省委、省政府“十项规定”，大力开展作风问题专项整治和突出问题整改，严厉查处各类违纪违法案件，坚决防止“四风”反弹，着力营造风清气正、崇廉尚实、干事创业、遵纪守法的良好政治生态。

各位代表！艰难困苦玉汝于成，奋起直追时不我待。我们坚信，任何压力都动摇不了我们迎难而上、勇毅笃行的坚定决心，任何困难都阻挡不了我们深化改革、专注发展的坚定步伐。让我们紧密团结在以习近平同志为总书记的党中央周围，在省委、省政府和市委的坚强领导下，紧紧依靠和团结全市人民，埋头苦干，砥砺前行，为建设开放、生态、法治、繁荣的川东北区域中心城市而努力奋斗！
